# Mutations in Nonessential eIF3k and eIF3l Genes Confer Lifespan Extension and Enhanced Resistance to ER Stress in *Caenorhabditis elegans*

**DOI:** 10.1371/journal.pgen.1006326

**Published:** 2016-09-30

**Authors:** Douglas J. Cattie, Claire E. Richardson, Kirthi C. Reddy, Elan M. Ness-Cohn, Rita Droste, Mary K. Thompson, Wendy V. Gilbert, Dennis H. Kim

**Affiliations:** 1 Department of Biology, Massachusetts Institute of Technology, Cambridge, Massachusetts, United States of America; 2 Howard Hughes Medical Institute, McGovern Institute for Brain Research, Massachusetts Institute of Technology, Cambridge, Massachusetts, United States of America; The University of North Carolina at Chapel Hill, UNITED STATES

## Abstract

The translation initiation factor eIF3 is a multi-subunit protein complex that coordinates the assembly of the 43S pre-initiation complex in eukaryotes. Prior studies have demonstrated that not all subunits of eIF3 are essential for the initiation of translation, suggesting that some subunits may serve regulatory roles. Here, we show that loss-of-function mutations in the genes encoding the conserved eIF3k and eIF3l subunits of the translation initiation complex eIF3 result in a 40% extension in lifespan and enhanced resistance to endoplasmic reticulum (ER) stress in *Caenorhabditis elegans*. In contrast to previously described mutations in genes encoding translation initiation components that confer lifespan extension in *C*. *elegans*, loss-of-function mutations in *eif-3*.*K* or *eif-3*.*L* are viable, and mutants show normal rates of growth and development, and have wild-type levels of bulk protein synthesis. Lifespan extension resulting from EIF-3.K or EIF-3.L deficiency is suppressed by a mutation in the Forkhead family transcription factor DAF-16. Mutations in *eif-3*.*K* or *eif-3*.*L* also confer enhanced resistance to ER stress, independent of IRE-1-XBP-1, ATF-6, and PEK-1, and independent of DAF-16. Our data suggest a pivotal functional role for conserved eIF3k and eIF3l accessory subunits of eIF3 in the regulation of cellular and organismal responses to ER stress and aging.

## Author Summary

The translation initiation factor eIF3 is the largest and most complex of the eukaryotic initiation factors, comprising 13 subunits in metazoans, and this protein complex plays a critical role in scaffolding key interactions among multiple cofactors required for translation initiation. Both evolutionary and biochemical evidence demonstrate that the critical functionality of this complex can be mediated by a limited repertoire of eIF3 subunits, suggesting regulatory or modulatory roles for the remaining subunits. Here, we show that two conserved subunits of this complex, eIF3k and eIF3l, are nonessential for normal growth and viability in *C*. *elegans*, and that in their absence, rates of bulk protein translation are not diminished. Deletion of either eIF3k or eIF3l confers a dramatic extension in lifespan and also improves protein-folding homeostasis of the endoplasmic reticulum in *C*. *elegans*. These data suggest that eIF3k and eIF3l may function in the regulation of cellular and organism physiology.

## Introduction

The genetic study of longevity of *C*. *elegans* has established how single mutations in conserved signaling pathways may have dramatic effects on animal lifespan [1,2]. In addition, reduction-of-function mutations or RNAi-mediated knockdown of genes encoding components required for mRNA translation, which reduces levels of protein synthesis and reduces rates of growth and development, have also been shown to extend lifespan of *C*. *elegans* [3–7]. Alterations in mRNA translation can also influence the expression of genes that may contribute to changes in lifespan [8,9], suggesting that lifespan extension is not simply a consequence of diminished levels of bulk mRNA translation when translation initiation is perturbed.

The regulation of mRNA translation is pivotal in a number of diverse responses to cellular stress. In particular, the accumulation of misfolded proteins in the endoplasmic reticulum (ER) activates a conserved compensatory response, the Unfolded Protein Response (UPR), which results in the increased expression of ER chaperones, components of ER-associated protein degradation, and attenuated translation through the phosphorylation of eIF2α [10]. The UPR was initially characterized with toxins that cause misfolded protein accumulation in the ER, but physiological roles of the UPR are now well established in the development of secretory cell types and in the pathogenesis of disease [10,11]. In *C*. *elegans*, the activation of innate immunity induces the UPR, which is required for survival in the presence of pathogenic bacteria [12]. A number of studies suggest that ER homeostasis and UPR activation may have both cell-autonomous and cell-non-autonomous effects on organismal stress physiology and longevity [13–16]. In addition, the NRF2-type transcription factor SKN-1 in *C*. *elegans*, an established regulator of longevity and stress resistance [17–19], is a focal point of key reciprocal regulatory interactions with UPR signaling pathways [8,20]. These data suggest that the maintenance of ER homeostasis is an important determinant of organismal stress response and longevity.

Here, we report the genetic characterization of subunits of the translation initiation factor eIF3, a 13-subunit complex that coordinates the assembly of the 43S pre-initiation complex that is competent for mRNA recruitment and translation initiation in eukaryotes [10,21]. Mapping of eIF3 subunit interactions by mass spectrometry [22] and recent cryo-electron microscopy structures [23,24] of the eIF3 complex have defined the configuration of eIF3 protein subunits and their interaction with the 40S ribosomal subunit. Whereas the 13 subunits of eIF3 are conserved from *C*. *elegans* to humans [25], eIF3 of *Saccharomyces cerevisiae* has only six subunits, and reconstitution of human eIF3 subunits in ribosome-toeprinting assays suggest that some eIF3 subunits, including eIF3k and eIF3l, may be dispensable for initiation of mRNA translation [26]. Of note, altered expression of eIF3 subunits have been observed to be associated with malignant transformation of mammalian cells [27,28]. Moreover, recent work utilizing RNA crosslinking and immunoprecipitation methods has shown that some mammalian eIF3 subunits associate with distinct mRNA transcripts involved in cellular proliferation [29]. Taken together, these prior studies of eIF3 suggest that the eIF3 complex may have dual roles—an essential functional core complex of eIF3 that is required for ribosome recruitment and initiation of mRNA translation, and an additional regulatory role that may modulate the differential translation of specific mRNAs or perhaps function outside the context of translation initiation.

Here, we report our studies that establish that eIF3 subunits *eif-3*.*K* and *eif-3*.*L* are nonessential in *C*. *elegans*, and that their loss does not affect rates of bulk protein synthesis. We find that loss of either subunit confers a 40% increase in lifespan and enhanced resistance to ER stress. Our data suggest that the evolutionarily conserved but nonessential *eif-3*.*K* and *eif-3*.*L* subunits of eIF3 function in the regulation of cellular ER homeostasis and organismal longevity.

## Results

### Mutations in *eif-3*.*K* and *eif-3*.*L* confer enhanced resistance to ER stress, independent of XBP-1

**Fig 1:**
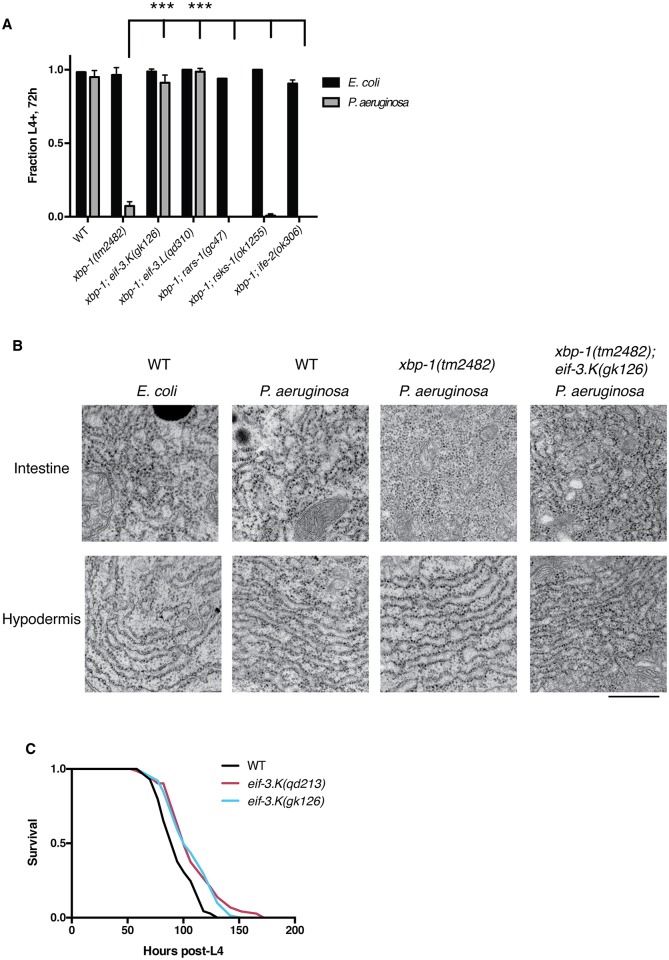
Loss of *eif-3*.*K* or *eif-3*.*L* suppresses larval lethality of *xbp-1* mutants on *P*. *aeruginosa*. (A) Development assay monitoring the growth and viability of the indicated genotypes on *E*. *coli* or *P*. *aeruginosa* at 25°C. 50–100 eggs were laid on each plate and following 72h the fraction reaching the L4 larval stage or older were counted. Error bars reflect the S.D. of 3 plates. A Student’s *t*-test was used to assess significance: **P<0.01, ***P < 0.001. (B) Transmission electron microscopy of L3 larvae cultivated on *E*. *coli* or *P*. *aeruginosa* at 60,000x magnification visualizing intestinal or hypodermal ER morphology. Scale bar, 500nm. (C) Survival curves of L4 larvae of the indicated genotypes at 25°C following transfer to plates containing *P*. *aeruginosa*. Two biological replicates were performed with similar results.

Previously, we demonstrated that the UPR is induced in the intestine of *C*. *elegans* in response to the activation of innate immunity following infection by pathogenic *P*. *aeruginosa* PA14 [12]. Activity of the UPR regulator XBP-1 was found to be essential for larval development on pathogenic *P*. *aeruginosa* but not on non-pathogenic *E*. *coli*, indicating an essential function for the UPR in the physiological tolerance of innate immune activation. In the current study, we performed a forward genetic screen to isolate mutations that could suppress the larval lethality of *xbp-1* mutant animals grown in the presence of *P*. *aeruginosa*. We identified one such suppressor mutation in the gene encoding the translation initiation factor subunit *eif-3*.*K*, *qd213*, which causes an early nonsense mutation in this gene (S1 Fig). We confirmed that another allele of *eif-3*.*K*, *gk126*, which contains a deletion that eliminates the start codon and is a putative null allele, also suppressed the larval lethality of the *xbp-1* mutant animals in the presence of *P*. *aeruginosa* (Fig 1A).

We previously noted that *xbp-1* mutants exposed to pathogenic *P*. *aeruginosa* exhibit changes in ER morphology in intestinal cells—in particular, the loss of normal sheet and tubular architecture with dilated luminal spaces consistent with chronic unmitigated ER stress, as visualized by transmission electron microscopy [12]. By contrast, no such changes in ER morphology were evident in neighboring hypodermal cells in *xbp-1* mutant animals in the presence of *P*. *aeruginosa* (Fig 1B). We observed that mutation of *eif-3*.*K* partially suppressed the aberrant rough-ER morphology of intestinal cells, suggesting that eif-3.K deficiency suppresses lethality by protecting against intestinal ER toxicity of the *xbp-1* mutant animals on *P*. *aeruginosa*.

We considered that mutations that eliminate *eif-3*.*K* function might suppress the larval lethality of *xbp-1* mutant animals grown on *P*. *aeruginosa* either by enhancing resistance to ER stress, or by diminishing the innate immune response, as we had previously observed with mutations in the *pmk-1* gene encoding the innate immune regulator p38 mitogen-activated protein kinase (MAPK) [12]. If a mutation in *eif-3*.*K* attenuated the innate immune response, then we would anticipate that the *eif-3*.*K* mutant would exhibit enhanced susceptibility to killing by *P*. *aeruginosa*. However, we found that *eif-3*.*K* mutant animals were in fact slightly resistant to *P*. *aeruginosa* infection (Fig 1C).

### *eif-3*.*K* and *eif-3*.*L* are not essential for viability or bulk protein synthesis in *C*. *elegans*

**Fig 2:**
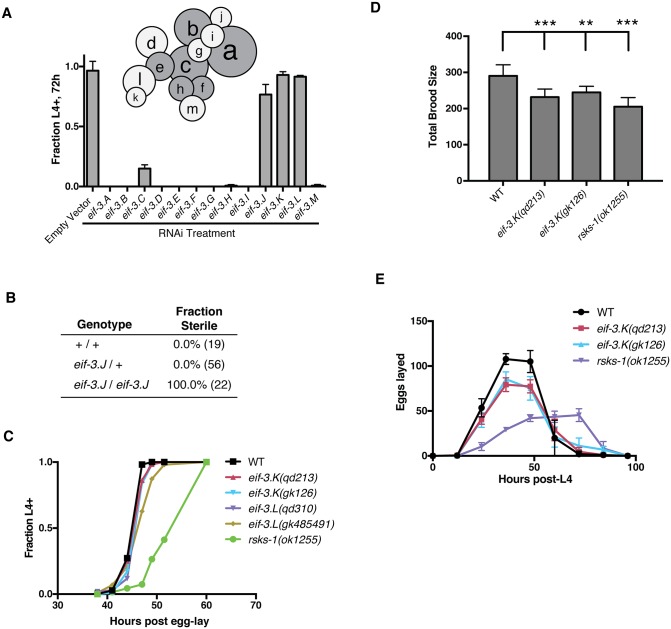
*eif-3*.*K* and *eif-3*.*L* are the only nonessential subunits in *C*. *elegans*, and mutants lacking these subunits do not have phenotypes associated with attenuated protein translation. (A) Individual eIF3 subunits were knocked down for one generation by RNAi feeding at 20°C. Eggs were then laid on the normal laboratory food source *E*. *coli* OP50 and evaluated for their ability to reach the larval stage L4 or older after 72h at 20°C. Inset: Interaction diagram of eIF3 subunits based on mass spectrometry of Zhou et al. [22]. Gray subunits represent the functional core of the mammalian complex sufficient to allow for initiation *in vitro* by Masutani et al. [26]. (B) Progeny of *eif-3*.*J* heterozygotes were assayed for sterility and then genotyped. Number of worms scored for each genotype is indicated in parentheses. (C) Developmental time course of the indicated genotypes. Populations were synchronized by egg lay and monitored over time for their development to the L4 larval stage or older. (D) Brood size of the indicated genotypes at 20°C. Error bars represent the S.D. of 10 animals. (E) Egg-laying rate was determined by transferring worms to new *E*. *coli* plates in 12h periods, and progeny were counted following 24h incubation at 20°C. Error bars represent the S.D. of 10 animals.

We were surprised to observe that *eif-3*.*K* is dispensable in *C*. *elegans*, as an ortholog of this eIF3 subunit is present in the genome of many metazoans, plants, and fungi within the eukaryotic phylogeny [25], suggesting an important function for this subunit. In order to understand the requirement for the other eIF3 subunits *in vivo*, we systematically knocked down each subunit by RNAi feeding and determined that almost all were required for normal growth and viability (Fig 2A), which corroborates prior genome-wide RNAi-based studies [30,31]. Knockdown of *eif-3*.*J* by RNAi was well-tolerated, but we generated a loss-of-function mutation in this gene, *eif-3*.*J (qd311)*, and observed that hermaphrodites homozygous for this mutation were sterile (Fig 2B). Knockdown of subunits *eif-3*.*K* and *eif-3*.*L*, by contrast, had no such effects on viability and fertility, and mutants carrying loss-of-function mutations in these genes are viable. This indicates that *eif-3*.*K* and *eif-3*.*L* are the only nonessential subunits of the eIF3 complex in *C*. *elegans*.

Biochemical analysis of the eIF3 complex in the filamentous fungus *Neurospora crassa* has shown that eIF3k and eIF3l form a dimer that then assembles with the rest of the complex [25]. This is consistent with the aforementioned structural studies of eIF3 in which these two subunits are physically associated with each other on the periphery of the complex [22,23], with eIF3k making almost all of its molecular contacts with eIF3 through the subunit eIF3l (Fig 2A, inset). These data suggest that any perturbation to the eIF3 complex in *C*. *elegans* generated by the absence of *eif-3*.*K* will be recapitulated by loss of *eif-3*.*L*, and phenotypes common to these two mutants likely reflect the function of these two genes within the context of the eIF3 complex. Consistent with this expectation, we observed that loss-of-function mutations in *eif-3*.*L* were able to suppress the larval lethality of the *xbp-1* mutant when exposed to *P*. *aeruginosa* (Fig 1A, S1 Fig).

We initially hypothesized that loss of these eIF3 subunits might promote resistance to ER stress by attenuating bulk protein synthesis, which would diminish the secretory load to the ER. However, we observed that loss-of-function mutations in each of three genes required for normal rates of protein synthesis, *rars-1*, *rsks-1*, and *ife-2* [5,6,32], were insufficient to suppress the larval lethality of *xbp-1* on *P*. *aeruginosa* (Fig 1A). Additionally, normal growth and larval developmental rate was observed in animals carrying loss-of-function mutations in *eif-3*.*K* and *eif-3*.*L* (Fig 2C), in contrast to the slowed growth rate of a mutant lacking the ribosomal protein S6 kinase *rsks-1*, which is known to have a decreased growth rate caused by attenuated protein synthesis [5]. We observed that the total brood size of *eif-3*.*K* mutants was diminished to ~ 80% of the wild-type brood size (Fig 2D). However, the age of peak egg-laying rate and the reproductive period of the *eif-3*.*K* mutant is identical to that of wildtype (Fig 2E), in marked contrast to that of the slow-growing *rsks-1* mutant.

**Fig 3:**
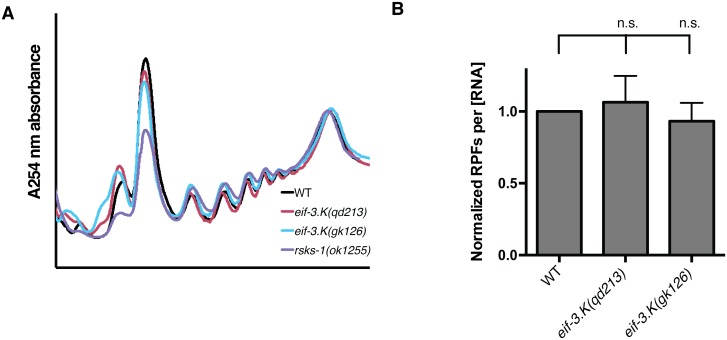
*eif-3*.*K* mutants do not have attenuated bulk translation. (A) Polysome profiles of the indicated genotypes, harvested at the L4 stage. Identical quantities of total RNA were loaded onto sucrose gradients, and absorbance was measured at 254 nm. (B) Relative translation assayed by ribosome profiling, using whole yeast lysates as an internal standard. Whole lysates from *S*. *cerevisiae* were added to whole *C*. *elegans* lysates at a ratio of 1:20 based on total RNA concentration. Following ribosome profiling, the relative abundance of ribosome protected fragments (RPF) was determined by dividing the total number of reads that map unambiguously to the *C*. *elegans* genome by those that map unambiguously to the *S*. *cerevisiae* genome. Raw counts are presented in S1 Table. Statistical significance was assessed by the Student’s *t*-test.

We observe that polysome profiles of *eif-3*.*K* mutants were superimposable on the corresponding profiles of wildtype animals, with a similar fraction of RNA sedimenting in the 60S, monosome, and polysome fractions (Fig 3A), suggesting that rates of bulk translation initiation are not diminished in these mutants lacking EIF-3.K or EIF-3.L. By contrast, the polysome of the *rsks-*1 mutant is skewed towards the polysome fraction and away from the 40S/60S/monosome fraction, consistent with a defect in this mutant in translational elongation [33].

In order to assess rates of bulk protein synthesis on a quantitative basis, we performed ribosome profiling of wildtype and two alleles of *eif-3*.*K*, including an internal standard for normalization. To enable relative quantitation, a known quantity of whole yeast lysate was added to whole worm lysate, and following ribosome protected fragment (RPF) isolation and sequencing, this internal standard allowed us to determine relative rates of protein translation among genotypes by counting the total number of footprints that map unambiguously to the *C*. *elegans* genome and normalizing by the number of footprints that map unambiguously to the *S*. *cerevisiae* genome. We observe that by this quantitative biochemical method there is no attenuation of bulk protein synthesis in the *eif-3*.*K* mutant (Fig 3B). We performed this experiment with the intent of identifying genes whose translational efficiency (TE) is suppressed or enhanced in the *eif-3*.*K* and *eif-3*.*L* mutant backgrounds, but with the exception of the genes *eif-3*.*K* and *eif-3*.*L* themselves, were unable to identify statistically significant and reproducible deviations in footprint and total mRNA abundance, though we cannot exclude the possibility of translational changes to lowly expressed genes.

### Mutations in *eif-3*.*K* and *eif-3*.*L* confer lifespan extension that is suppressed by a mutation in DAF-16

**Fig 4:**
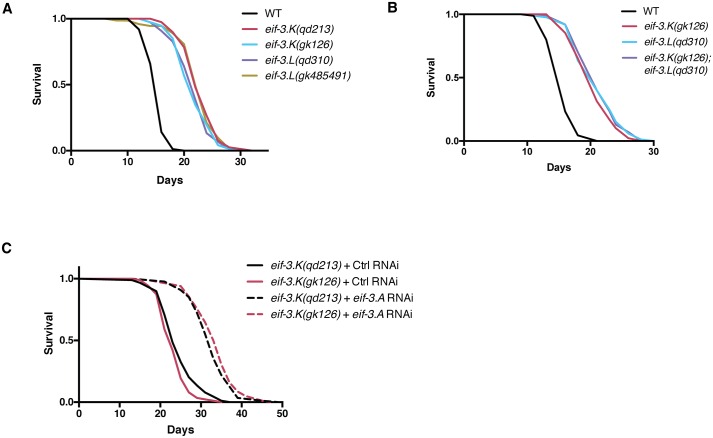
Loss of *eif-3*.*K* or *eif-3*.*L* confers lifespan extension in *C*. *elegans* in a manner distinct from depletion of essential eIF3 subunits. (**A-C**) Survival curves of the indicated genotypes at 25°C. Times indicated are days post-L4 stage. Lifespan statistics and replicate data is presented in S2 Table. For RNAi lifespan experiments, survival curves of the indicated genotypes transferred to control (*gfp*) or *eif-3*.*A* RNAi bacteria as L4 larvae. Worms were grown at 25°C for 3 days and shifted to 20°C for the remainder of the experiment.

Strikingly, we observed a 40% increase in longevity among mutants lacking the nonessential eIF3 subunits *eif-3*.*K* and *eif-3*.*L* (Fig 4A). Prior studies have shown that molecular and genetic reduction of the levels of proteins required for mRNA translation, such as ribosomal proteins or initiation factors, is sufficient to extend lifespan [3–7,9,34–36], but in such instances bulk translation is diminished and the organism has a correspondingly slowed rate of growth and development. We did not observe a synergistic increase in longevity in a double mutant strain carrying mutations in both *eif-3*.*K* and *eif-3*.*L* (Fig 4B), indicating that in the wild-type background these two genes cooperate for their normal biological function.

RNAi-mediated depletion of essential eIF3 subunits *eif-3*.*A (egl-45)*, *eif-3*.*B*, and *eif-3*.*F* have previously been identified as a means of extending lifespan in *C*. *elegans* through the attenuation of protein synthesis [4]. We observed that RNAi of the essential eIF3 subunit *eif-3*.*A (egl-45)* could further increase the lifespan of the *eif-3*.*K* mutants (Fig 4C), consistent with the idea that loss of EIF-3.K and EIF-3.L subunits promotes longevity in a manner that is distinct from mechanisms caused by depletion of essential eIF3 subunits. The additive nature of these interventions on longevity suggests that loss of nonessential eIF3 subunits and depletion of essential eIF3 subunits contribute to lifespan extension through independent mechanisms.

**Fig 5:**
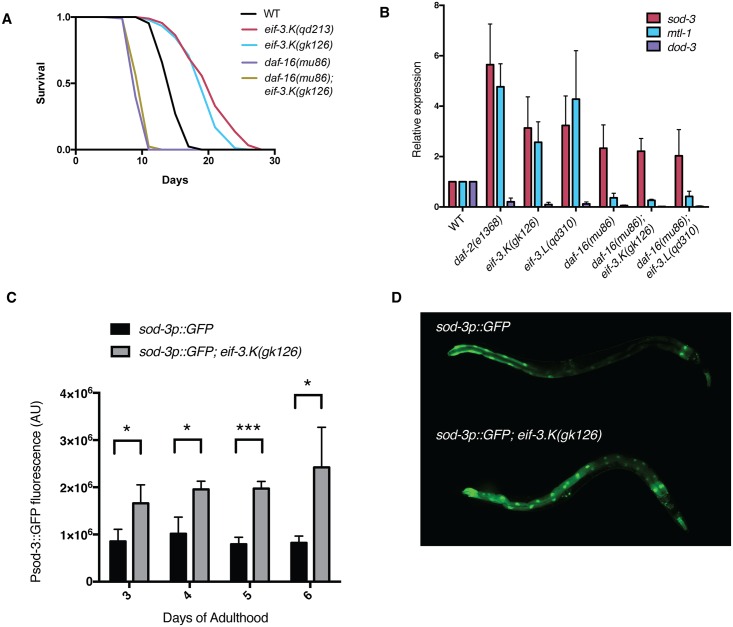
Loss of *eif-3*.*K* or *eif-3*.*L* hyper-activates the DAF-16 transcription factor, which is required for lifespan extension. (A) Survival curves of the indicated genotypes at 25°C. Times indicated are days post-L4 stage. Lifespan statistics and replicate data is presented in S2 Table. (B) Relative expression of *sod-3*, *mtl-1*, and *dod-3* in the indicated genetic backgrounds as assayed by qRT-PCR of synchronous L4 larvae. Error bars represent the S.D. of three replicates. (C) Total fluorescence of the *sod-3p*::*GFP* transgene in worms of the indicated genotypes was quantitated at several adult timepoints. Error bars represent the S.D. of 10 worms. (D) Composite image depicting representative images of Day 3 adult worms of each indicated genotype expressing the transgene *sod-3p*::*GFP*.

To gain insight into the downstream mechanisms involved in lifespan extension conferred by loss of EIF-3.K, we carried out genetic epistasis analysis with the *eif-3*.*K* mutant. We determined that a mutation in the Forkhead transcription factor DAF-16 completely suppressed the lifespan extension conferred by mutation of *eif-3*.*K* (Fig 5A), which suggests that DAF-16 functions downstream of, or in parallel to, the loss of *eif-3*.*K* or *eif-3*.*L* in modulating organismal longevity of *C*. *elegans*. In order to determine if activity of the DAF-16 transcription factor is modulated in the *eif-3*.*K/L* mutant backgrounds, we performed qRT-PCR on well-characterized DAF-16 targets, including two known to be upregulated by mutation in *daf-2* (*sod-3 and mtl-1*), as well as one known to be downregulated in *daf-2* mutants (*dod-3*) [37]. We found that the relative expression of these genes in the *daf-2* mutant background was recapitulated by loss of *eif-3*.*K* or *eif-3*.*L* (Fig 5B), indicative of increased DAF-16 activity in the absence of *eif-3*.*K* or *eif-3*.*L*. Expression of these DAF-16 target genes in the *daf-16; eif-3*.*K* and *daf-16; eif-3*.*L* double mutants was not appreciably different from that of the *daf-16* single mutant, indicating that the changes in expression of these genes in the *eif-3*.*K* and *eif-3*.*L* mutants is dependent on DAF-16. We also observed that fluorescence from a *sod-3p*::*GFP* transgene was two-fold higher in the *eif-3*.*K* mutant background, and that this increased fluorescence remains steady throughout early adulthood (Fig 5C). Fluorescence from this transgene was particularly increased within intestinal cells (Fig 5D), where DAF-16 activity has been shown to be particularly important in promoting lifespan extension [38]. Together these data suggest that increased activity of DAF-16 contributes to the lifespan extension observed in *eif-3*.*K* and *eif-3*.*L* mutant animals, consistent with our observed epistasis data (Fig 5A).

### Mutations in *eif-3*.*K* and *eif-3*.*L* confer resistance to tunicamycin

**Fig 6:**
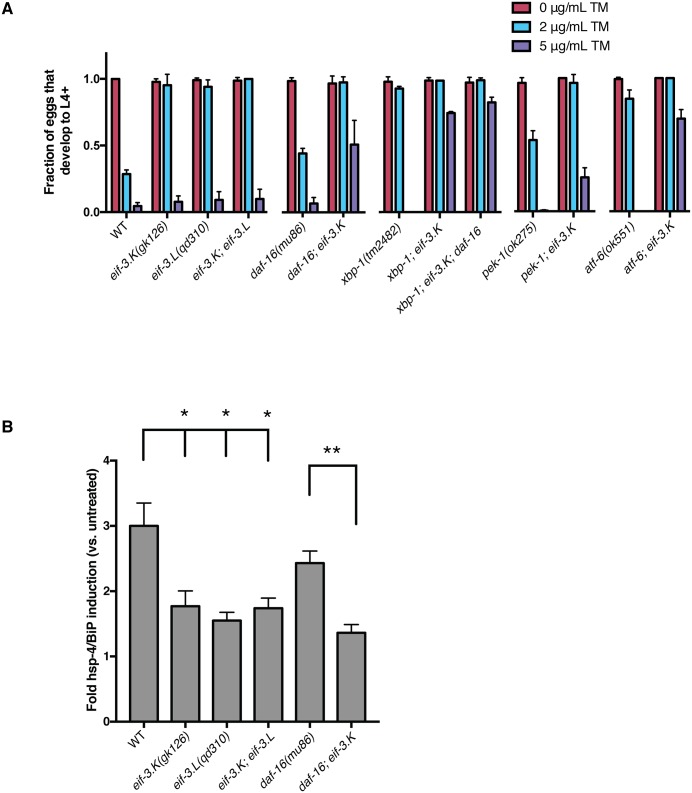
Mutants lacking *eif-3*.*K* or *eif-3*.*L* are resistant to tunicamycin, independent of *daf-16* and regulators of the Unfolded Protein Response. (A) Animals were scored for their ability to reach the L4 stage or older 72h after 50–100 eggs were laid on plates containing 0, 2, or 5 μg/mL tunicamycin. Error bars reflect the S.D. of three plates. Two biological replicates were performed with similar results. (B) *hsp-4* induction was measured by qRT-PCR after L4 larvae were transferred to plates containing 10 μg/mL tunicamycin for 4h. Error bars reflect the SEM of three replicates. A Student’s *t*-test was used to assess significance: *P < 0.05, **P < 0.01.

The observation that the loss of *eif-3*.*K* or *eif-3*.*L* could suppress the larval lethality and corresponding disruption of ER morphology of *xbp-1* mutant animals exposed to *P*. *aeruginosa* suggested that *eif-3*.*K* or *eif-3*.*L* mutants might exhibit enhanced resistance to ER stress. We assayed *eif-3*.*K* and *eif-3*.*L* mutants for their sensitivity to the ER-toxic drug tunicamycin, which inhibits protein glycosylation and results in protein accumulation in the ER. At a concentration of tunicamycin (2 μg/mL) at which the majority of wild type animals arrest and die during larval development, *eif-3*.*K* and *eif-3*.*L* mutants were resistant to this treatment (Fig 6A). Furthermore, we observed that *daf-16* was not required for resistance to tunicamycin, as the *daf-16; eif-3*.*K* double mutant was more resistant to tunicamycin than the *daf-16* single mutant. We wondered whether the resistance of the *eif-3*.*K* and *eif-3*.*L* mutants might involve some compensatory function from the other branches of the unfolded protein response, yet we find that none of the UPR regulators–*xbp-1*, *pek-1*, or *atf-6* –are required for the tunicamycin resistance of the *eif-3*.*K* mutant. These data indicate that, unlike lifespan extension, the improved ER homeostasis in these mutants is DAF-16 independent, and is additionally independent of the UPR.

We next assayed the induction of the ER-resident folding chaperone *hsp-4/BiP* upon acute tunicamycin treatment, which is a reporter of unfolded protein response (UPR) activation. We observed that *eif-3*.*K* and *eif-3*.*L* mutants exhibited reduced induction of *hsp-4/BiP* mRNA at high concentrations of tunicamycin (Fig 6B), and that this trend was also observed in the absence of *daf-16*. At first glance, this might appear somewhat paradoxical, as the wild type strain that has more susceptibility to tunicamycin has an increased induction of protective chaperone expression, but we suggest that these data reflect an enhancement to the ER folding capacity in the *eif-3*.*K* mutant that is capable of remediating stress produced by acute tunicamycin exposure. This phenomenon of improved tunicamycin resistance despite decreased *hsp-4* induction has previously been observed in *daf-2* mutants, but in a context that is *daf-16*-dependent [14].

### EIF-3.K is expressed ubiquitously in *C*. *elegans*

**Fig 7:**
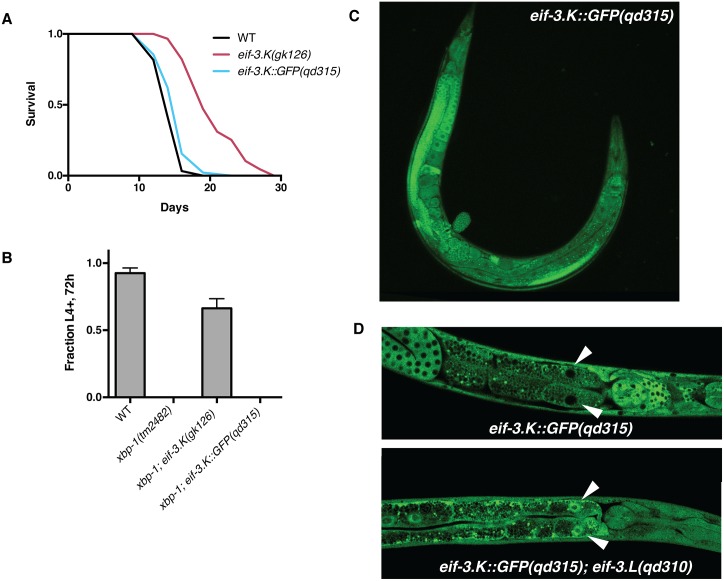
EIF-3.K::GFP is ubiquitously expressed in *C*. *elegans* and is localized to the cytosol. (A) Lifespan curves of wild-type, the *eif-3*.*K* mutant, and a strain containing a fluorescently-tagged allele of *eif-3*.*K*. (B) Developmental assay monitoring the growth and viability of the indicated genotypes on *P*. *aeruginosa* at 25°C. Error bars reflect the S.D. of 3 plates. (C) Fluorescent micrograph of EIF-3.K::GFP at 10x magnification. (D) Fluorescent confocal image depicting intestinal nuclei of L4 larvae of the indicated genotypes at 20x magnification. Arrows indicate intestinal nuclei.

Given that subunits *eif-3*.*K* and *eif-3*.*L* are nonessential, and given the apparent bias towards retention of these two genes in multicellular eukaryotes [25], we wondered whether expression of these genes might be restricted to specific tissues. To this end, we engineered a C-terminal GFP tag onto the endogenous locus of *eif-3*.*K* using CRISPR/Cas-9, and validated function of this gene by phenotypic analysis. The fluorescently-tagged allele was neither long-lived (Fig 7A) nor able to suppress the larval lethality of the *xbp-*1 mutant grown on *P*. *aeruginosa* (Fig 7B), suggesting that the fusion-protein retained wild-type function. We find that EIF-3.K::GFP is expressed in all tissues, with especially bright fluorescence in the *C*. *elegans* germline (Fig 7C). As expected, expression is restricted to the cytosol, the site of translation initiation (Fig 7D).

We observed that mutation of *eif-3*.*L* in the strain carrying the EIF-3.K::GFP permits the diffusion of EIF-3.K::GFP into the nucleus, though nucleolar exclusion is maintained (Fig 7D). This observation is consistent with the crystallographic and mass spectrometric evidence that *eif-3*.*L* serves as a bridge between *eif-3*.*K* and the rest of the eIF3 complex.

We performed tissue-specific rescue of the *eif-3*.*K* mutant in an effort to determine the specific tissues in which *eif-3*.*K* activity influences lifespan and ER stress resistance. We observed partial rescue of the lifespan extension phenotype of the *eif-3*.*K* mutant when *eif-3*.*K* was expressed under the control of multiple different tissue-specific promoters, including those directing expression in the muscle, intestine, and nervous system (S2A Fig), suggesting that the downstream consequence of EIF-3.K function in multiple tissues contributes to the modulation of longevity. We also performed tissue-specific rescue of *eif-3*.*K* in the *xbp-1; eif-3*.*K* mutant and evaluated these transgenic animals for their ability to develop on *P*. *aeruginosa* (S2B Fig). We anticipated that *eif-3*.*K* functions cell autonomously in the intestine to regulate ER homeostasis, and consistent with this expectation, we observed that intestinal expression of *eif-3*.*K* could rescue larval lethality in *xbp-1; eif-3*.*K* animals. Unexpectedly, we observed that neuronal expression of *eif-3*.*K* in *xbp-1; eif-3*.*K* animals was also able to restore larval lethality. However, we note that an important caveat in the interpretation of these experiments involving the heterologous overexpression of *eif-3*.*K* in specific tissues is potential toxicity that might diminish survival in lifespan and larval development assays.

## Discussion

Whereas multiple studies have established connections between the knockdown or loss of translation initiation factors and ribosome-associated proteins with improved longevity, our data demonstrate that loss of two conserved subunits of the eIF3 complex, EIF-3.K and EIF-3.L, confers extension in lifespan without effects on bulk translation and corresponding effects on rates of growth and development. We have also determined that loss of EIF-3.K or EIF-3.L also confers enhanced resistance to ER stress, both in growth and development on tunicamycin, as well as in *xbp-1* animals exposed to *P*. *aeruginosa*. Our genetic analysis suggests that lifespan extension is dependent on DAF-16, whereas loss of EIF-3.K and EIF-3.L confers enhanced resistance to ER stress independent of DAF-16, thus suggesting that distinct mechanisms are involved in conferring lifespan extension and ER stress resistance in *eif-3*.*K* and *eif-3*.*L* mutant animals. Moreover, our data suggest that EIF-3.K and EIF-3.L promote enhanced resistance to pharmacological and physiological ER stress independent of the three arms of the Unfolded Protein Response mediated by IRE-1-XBP-1, PEK-1, and ATF-6.

A number of studies point to roles for the eIF3 complex in the post-transcriptional regulation of gene expression. *Schizosaccharomyces pombe*, for example, possess two distinct eIF3 complexes, which are distinguished by the presence of either the eIF3e or eIF3m subunits, and the eIF3e-containing complex translates only a very limited set of mRNAs [39]. In zebrafish, a novel isoform of eIF3h is expressed only in the eyes and nervous system and guides development of these tissues through the translational regulation of a subset of mRNAs [40,41]. Most recently, the mammalian eIF3 complex was found to bind N6-methyladenosine residues within the 5’ UTR of some mRNAs in order to enhance their translation, including the heat shock protein HSP70, through a cap-independent mechanism [42]. We have also not excluded an alternative hypothesis in which EIF-3.K and EIF-3.L might regulate cellular physiology though pathways outside of the eIF3 complex and mRNA translation. For example, the eIF3k subunit itself has been implicated in a caspase-dependent apoptosis-promoting function [43,44]. Nevertheless, our favored model, in view of the privileged position of EIF-3.K and EIF-3.L in close proximity to the translational apparatus, is that these nonessential but conserved accessary subunits of eIF3 may influence the physiology of aging and ER homeostasis through interactions with the eIF3 complex that modulate the differential translation of mRNAs. While in the current study we were unable to identify reproducible changes to the translational efficiency of individual mRNAs in mutants lacking these subunits by analyzing whole animals at basal conditions, we suspect that translational changes may still be occurring in perhaps a subset of tissues, possibly in response to a stressor or condition which has not yet been experimentally tested.

## Materials and Methods

### Strains and genetics

*C*. *elegans* were cultured on OP50 as described [45]. The following strains were generated in the lab through mutagenesis or obtained from the *Caenorhabditis Genetics Center*: N2 (Bristol), ZD891 *eif-3*.*K(qd213)*, ZD892 *eif-3*.*K(gk126)*, ZD1258 *eif-3*.*L[C17G10*.*9(qd310)]*, ZD1098 *eif-3*.*L[C17G10*.*9(gk485491))*, ZD1828 *eif-3*.*K(gk126); eif-3*.*L(qd310)*, ZD1364 *rsks-1(ok1255)*, ZD1022 *daf-16(mu86)*, ZD1036 *daf-16(mu86); eif-3*.*K(gk126)*, ZD418 *xbp-1(tm2482)*, ZD613 *xbp-1(tm2482); agIs219 [T24B8*.*5*:*GFP*:*unc-54-3' UTR]; eif-3*.*K(qd213)*, ZD893 *xbp-1(tm2482); eif-3*.*K(gk126)*, ZD1085 *xbp-1(tm2482); rars-1(gc47)*, ZD990 *xbp-1(tm2482); rsks1(ok1255)*, ZD988 *xbp-1(tm2482); ife-2(ok306)*, RB772 *atf-6(ok551)*, ZD1252 *atf-6(ok551); eif-3*.*K(gk126)*, MC366 *pek-1(ok275)*, ZD1253 *pek-1(ok275); eif-3*.*K(gk126)*, ZD1829 *xbp-1(tm2482); eif-3*.*K(gk126); daf-16(mu86)*, ZD1292 *hIn1 unc-54(h1040) / eif-3*.*J[Y40B1B*.*5(qd311)] I*, ZD1421 *eif-3*.*K(qd315[eif-3*.*K*:*2xTY1*:*GFP*:*3xFLAG])*, ZD1422 *eif-3*.*K(qd315[eif-3*.*K*:*2xTY1*:*GFP*:*3xFLAG]); eif-3*.*L[C17G10*.*9(qd310)]*.

### Mutation of *eif-3*.*J* by CRISPR/Cas9

A loss-of-function allele of *eif-3*.*J (Y40B1B*.*5)* was generated as previously described [46,47]. The gRNA was constructed using the pRB1017 backbone, targeting the sequence agccgctccaacattcgccatgg, which occurs within the first 160bp of the CDS. This gRNA was injected at a concentration of 45 ng/μL, along with the co-injection marker pCFJ90 at 2.5 ng/μL, eft-3p::Cas9::NLS::tbb-1 3’UTR at 50 ng/μL. Transgenic F1s were screened for deletion by Sanger sequencing, and an early nonsense allele was identified, designated *qd311*. As homozygotes for this mutation are sterile, this allele was balanced with the hIn1 LGI balanced chromosome.

### *P*. *aeruginosa* development assay

Worms of the indicated genotypes were egg-layed onto either 6 cm NGM plates seeded with *E*. *coli* OP50 or 3.5 cm Slow Kill Assay (SKA) plates seeded with *P*. *aeruginosa* PA14 as described [48]. For SKA plates, 7μL of overnight cultures of PA14 in LB were seeded onto the center of a 3.5 cm SKA plate. These plates were incubated at 37°C for 24h, and room temperature for 24h prior to use. Following egg-lay onto OP50 or PA14, plates were transferred to 25°C for 72h and then worms were scored based on their development to the L4 larval stage or older. Plates contained 50–100 eggs and three plates were averaged within each experiment.

### Transmission electron microscopy

Worms of the indicated genotypes were synchronized by hypochlorite treatment and grown on *E*. *coli* or *P*. *aeruginosa* at 25°C until the L3 larval stage, for about 23h. Worms were then fixed and imaged as described [49], using Standard Immersion Fixation. Images were acquired at 60,000x.

### *P*. *aeruginosa* survival assay

SKA plates were prepared as described above, but with the addition of 50 μgml^−1^ 5-fluoro-2’-deoxyuridine (FUDR) in order to suppress progeny production. 30 L4 worms were transferred to each SKA plate, incubated at 25°C, and scored every 12 hours for survival. Three plates were scored and combined per genotype in order to generate survival curves.

### RNAi knockdown of eIF3 subunits

Approximately 20 L4 worms were transferred to RNAi plates that were seeded with HT115 *E*. *coli* containing plasmids targeting the genes of interest as collected from the Ahringer RNAi library [30]. After 2 days at 16°C, gravid worms were transferred to OP50 plates and allowed to lay 50–100 eggs, in triplicate. Following 72h at 20°C, the fraction of worms reaching the L4 stage or older were counted. RNAi clones not present in the library (*eif-3*.*C* and *eif-3*.*M(cif-1)*), were constructed by ligating ~1kB of the genomic coding region into the empty vector L4440 followed by transformation into the *E*. *coli* strain HT115. RNAi plates consisted of NGM supplemented with 2mM isopropyl b-D-1 thiogalactopyranoside (IPTG) and 25 μg/mL carbenicillin.

### Developmental time-course

Worms of the indicated genotype were synchronized by egg laying, and assessed periodically for their development to the L4 stage or older. This assay was carried out at 20°C.

### Brood size assay

Worms were synchronized by egg-laying. Following 24h at 20°C, worms were transferred singly to plates containing *E*. *coli* OP50 every 12h for the duration of the egg-laying period. Following 24h, the progeny were counted. 10 worms were scored per genotype.

### Polysome profiling

Polysome profiling was carried out essentially as described [6,50], but with the following changes. Roughly ~100,000 worms were synchronized by bleaching and grown to the L4 stage. Worms were washed once in M9, and again in M9 + 0.1mg/mL cyclohexamide, before being flash frozen in liquid N_2_. Worm pellets along with 1mL lysis buffer (10 mM Tris-HCl, pH 7.4, 5mM MgCl2, 100mM KCl, 2mM dithiothreitol, 100μgml^−1^ cycloheximide, 1% Triton X-100, 500Uml^−1^ RNasin Plus, and protease inhibitor (1x complete, EDTA-free, Roche)), were lysed using 40 strokes on a dounce homogenizer. Lysates were cleared of debris by centrifugation (15 mins at 20,000g), and 25 OD_260_ units of lysate was loaded onto 10–50% sucrose gradients. Samples were spun for 2.75h at 35,000 rpm in a Beckman SW41 rotor, and profiles were generated using a BioComp gradient master.

### Ribosome profiling

Worm lysates were prepared as described above. Yeast lysates were prepared by growing *S*. *cerevisiae* strain FY2 to exponential growth phase (O.D. 600 of ~1.0) in 50 mL YPD medium at 30°C, pelleted at 2,000x*g* for 2 minutes, and resuspended in 3mL lysis buffer. Approximately 0.5g of 0.5mm glass beads was added to resuspended yeast and vortexted at maximum speed for 2 minutes. Lysates were then cleared of debris by centrifugation at top speed for 10 minutes at 4°C. Lysates were flash-frozen in liquid N_2_ until ready for use. To enable quantitation following ribosome profiling, 25 OD_260_ units of worm lysate was mixed with 1.25 OD_260_ units of yeast lysate, and ribosome footprinting was performed as described [51]. Following sequencing, libraries were aligned to both the *C*. *elegans* and *S*. *cerevisiae* genomes and total reads aligning to each genome were tabulated.

### Lifespan analysis

Lifespan assays were carried out as previously described [52]. Briefly, 30 L4 worms were transferred to NGM plates containing 50 μgml^−1^ 5-fluoro-2’-deoxyuridine (FUDR) in triplicate and the assay was carried out at 25°C. Worms were scored every 1–2 days for survival. For lifespan on RNAi bacteria, worms were transferred to RNAi plates containing 50 μgml^−1^ FUDR, and plates were shifted to 20°C after 3d in order to minimize explosion.

### qRT-PCR of DAF-16 targets

Approximately 2,000 synchronized L4 worms of the indicated genotypes were harvested in M9 buffer, washed in M9 to purge the intestine of bacteria, and flash frozen. RNA was isolated and qRT-PCR was performed as described [12]. Genes were normalized to the housekeeping gene *act-1*, and relative expression was calculated using the ΔΔCt method [53].

### Quantitation of *sod-3p*::*GFP* transgene

Synchronized worms of the indicated genotype were grown at 20°C, and were transferred to new plates every 24h to avoid starvation resulting from progeny production. Images were acquired with an Axioimager Z1 microscope using animals anaesthetized in 50 mM sodium azide. To quantify GFP fluorescence, animals were imaged at 10x magnification, and the total fluorescence of the animal was determined using FIJI software.

### Tunicamycin survival assay

Worms were synchronized by egg laying onto NGM plates containing 0, 2, or 5 μgml^−1^ tunicamycin. These plates were made using a 25 mg ml^−1^ stock of tunicamycin dissolved in DMSO, and seeded with *E*. *coli* OP50. After 72h, worms were evaluated for their development to the L4 larval stage or older.

### *hsp-4* induction assay:

Worms of the indicated genotype were synchronized by hypochlorite treatment and allowed to develop to the L4 stage on NGM plates. Approximately 2,000 worms per treatment were then washed onto new plates containing 10 μg/mL of tunicamycin for 4h. Worms were then harvested and RNA preparation and qRT-PCR were performed as described [12]. *hsp-4* was normalized to the housekeeping gene *act-1*, and each condition was performed in triplicate.

### Tagging of *eif-3*.*K* by CRISPR/Cas9

The endogenous locus of eif-3.K was tagged as previously described [46,47]. For the homologous repair template, a 2xTY1::GFP::3xFLAG tag was amplified from clone CBGtg9050D0789D from the TransgeneOme project [54], and was subsequently inserted in-frame into a plasmid containing 1.6 kB homology upstream of the eif-3.K stop codon and 1.1 kB homology downstream of the stop codon using Gibson assembly. The gRNA was constructed using the pRB1017 backbone, targeting the sequence gatattaaagagtcaacgg, which is less than 10 bp from the site of insertion.

### Generation of transgenic animals

The *eif-3*.*K* cDNA was amplified from wildtype cDNA by PCR. The *unc-54* 3’ UTR was amplified by PCR from Fire Vector pPD95.75. The promoters for *myo-3* (1.3 kb), *dpy-7* (1.3 kb), *ges-1* (2.9 kb), and *rab-3* (1.4 kb), were amplified from wild-type genomic DNA by PCR. DNA constructs (promoter::cDNA::*unc-54* 3’ UTR) were synthesized using Gibson Assembly and sequences were verified using Sanger sequencing. Genomic *eif-3*.*K* was amplified from fosmid clone WRM0624aG04 (Source BioScience), including 4.6 kb upstream and 1.3 kb downstream of the *eif-3*.*K* CDS. DNA constructs were injected into animals at a concentration of 25 ng/μl for rescue plasmids or 1 ng/μL for genomic PCR rescue construct, along with a plasmid carrying either *ges-1p*::*gfp* (25 ng/μL) or *ofm-1p*::*gfp* (50 ng/ml). At least three independent transgenic lines were analyzed for each rescue construct.

## Supporting Information

S1 Table Read counts mapped to *C*. *elegans* or *S*. *cerevisiae* genome from ribosome profiling experiment. (PDF)

S2 Table Statistics for lifespan experiments. (PDF)

S1 Fig Multiple alleles of *eif-3*.*K* and *eif-3*.*L* suppresses larval lethality of *xbp-1* mutants on *P*. *aeruginosa*. Development assay monitoring the growth and viability of the indicated genotypes on *E*. *coli* or *P*. *aeruginosa* at 25°C. 50–100 eggs were laid on each plate and following 72h the fraction reaching the L4 larval stage or older were counted. Error bars reflect the S.D. of 3 plates. A Student’s *t*-test was used to assess significance: **P<0.01, ***P < 0.001.
(PDF)

S2 Fig Tissue-specific rescue of *eif-3*.*K* for longevity and development on *P*. *aeruginosa*. (A) Survival curves of the indicated genotypes at 25°C. Times indicated are days post-L4 stage. (B) Development assay monitoring the growth and viability of the indicated genotypes on *P*. *aeruginosa* at 25°C. 50–100 eggs were laid on each plate and following 72h the fraction reaching the L4 larval stage or older were counted. Error bars reflect the S.D. of 3 plates.
(PDF)
