# Supplementary material for: Mutations in Nonessential eIF3k and eIF3l Genes Confer Lifespan Extension and Enhanced Resistance to ER Stress in Caenorhabditis elegans
Source: PLoS Genet. 2016 Sep 30;12(9):e1006326. doi: 10.1371/journal.pgen.1006326 (PMC5045169; doi:10.1371/journal.pgen.1006326)
Supplement: S1 Table — (PDF) [file pgen.1006326.s001.pdf]

Sample	*C. elegans* CDS reads	*S. cerevisiae* CDS reads	Ratio
WT replicate 1	10283353	574131	17.91
WT replicate 2	12581951	575114	21.88
eif-3.K(qd213) replicate 1	9829209	459678	21.38
eif-3.K(qd213) replicate 2	8902662	434957	20.47
eif-3.K(gk126) replicate 1	6870555	374967	18.32
eif-3.K(gk126) replicate 2	8286071	449641	18.43
