# Supplementary material for: Mutations in Nonessential eIF3k and eIF3l Genes Confer Lifespan Extension and Enhanced Resistance to ER Stress in Caenorhabditis elegans
Source: PLoS Genet. 2016 Sep 30;12(9):e1006326. doi: 10.1371/journal.pgen.1006326 (PMC5045169; doi:10.1371/journal.pgen.1006326)
Supplement: S2 Table — (PDF) [file pgen.1006326.s002.pdf]

Table S2.

Statistics for lifespan experiments.

***eif-3.K/eif-3.L* Longevity Experiments, *daf-16* epistasis**

Experiment	Genotype	Mean lifespan ± SEM	P value vs control (Log rank test)	Percent Increase vs. Control	Number of worms	Comments
1	N2	13.3 ± 0.2	Control	Control	83/90	Performed at 25°C
	eif-3.K(qd213)	19.7 ± 0.3	< 0.0001	48.1	80/90	
	eif-3.K(gk126)	19.6 ± 0.3	< 0.0001	47.4	81/90	
2	N2	14.8 ± 0.2	Control	Control	85/90	Performed at 25°C
	eif-3.K(qd213)	20.4 ± 0.4	< 0.0001	37.8	88/90	Presented in Figure 2C
	eif-3.K(gk126)	19.3 ± 0.3	< 0.0001	30.4	89/90	
	eif-3.L(gk485491)	19.3 ± 0.4	< 0.0001	30.4	87/90	
	daf-16(mu86)	9.8 ± 0.1	Control	Control	89/90	
	daf-16(mu86); eif-3.K(gk126)	10.2 ± 0.1	0.0132	4.1	85/90	
3	N2	15.4 ± 0.2	Control	Control	83/90	Performed at 25°C
	eif-3.K(qd213)	21.0 ± 0.3	< 0.0001	36.4	88/90	
	eif-3.K(gk126)	19.6 ± 0.4	< 0.0001	27.3	85/90	
	eif-3.L(gk485491)	20.6 ± 0.5	< 0.0001	33.8	78/90	
	daf-16(mu86)	9.9 ± 0.1	Control	Control	90/90	
	daf-16(mu86); eif-3.K(gk126)	9.8 ± 0.1	0.7288	-1.0	83/90	
4	N2	15.5 ± 0.2	Control	Control	85/90	Performed at 25°C
	eif-3.K(qd213)	23.0 ± 0.3	< 0.0001	48.4	79/90	Presented in Figure 2A
	eif-3.K(gk126)	21.9 ± 0.4	< 0.0001	41.3	72/90	
	eif-3.L(qd310)	21.9 ± 0.4	< 0.0001	41.3	75/90	
	eif-3.L(gk485491)	22.7 ± 0.5	< 0.0001	46.5	71/90	
5	N2	18.3 ± 0.6	Control	Control	48/90	Performed at 20°C
	eif-3.K(qd213)	26.1 ± 0.5	< 0.0001	42.6	78/90	
	eif-3.K(gk126)	25.3 ± 0.5	< 0.0001	38.3	85/90	
	daf-16(mu86)	11.7 ± 0.2	Control	Control	79/90	
	daf-16(mu86); eif-3.K(gk126)	12.5 ± 0.2	0.0156	6.8	86/90	
6	N2	21.3 ± 0.5	Control	Control	47/90	Performed at 20°C
	eif-3.K(qd213)	34.4 ± 0.9	< 0.0001	61.5	75/90	
	eif-3.K(gk126)	27.4 ± 0.6	< 0.0001	28.6	82/90	
	daf-16(mu86)	12.7 ± 0.2	Control	Control	76/90	
	daf-16(mu86); eif-3.K(gk126)	13.1 ± 0.2	0.1572	3.1	80/90	

RNAi Experiments

7	*eif-3.K(qd213) on GFP RNAi*	24.6 ± 0.5	Control	Control	89/90	Performed at 20°C
	eif-3.K(qd213) on eif-3.A RNAi	32.6 ± 0.5	< 0.0001	32.5	87/90	Presented in Figure 2B
	eif-3.K(gk126) on GFP RNAi	23.3 ± 0.4	Control	Control	88/90	
	eif-3.K(gk126) on eif-3.A RNAi	33.6 ± 0.6	< 0.0001	44.2	82/90	
8	*eif-3.K(qd213) on GFP RNAi*	24.3 ± 0.5	Control	Control	87/90	Performed at 20°C
	eif-3.K(qd213) on eif-3.A RNAi	35.0 ± 0.7	< 0.0001	44.0	80/90	
	eif-3.K(gk126) on GFP RNAi	22.6 ± 0.5	Control	Control	89/90	
	eif-3.K(gk126) on eif-3.A RNAi	33.3 ± 0.8	< 0.0001	47.3	89/90	
9	*eif-3.K(qd213) on GFP RNAi*	24.0 ± 0.4	Control	Control	89/90	Performed at 20°C
	eif-3.K(qd213) on eif-3.A RNAi	33.5 ± 0.7	< 0.0001	39.6	88/90	
	eif-3.K(gk126) on GFP RNAi	24.2 ± 0.4	Control	Control	90/90	
	eif-3.K(gk126) on eif-3.A RNAi	34.8 ± 0.7	< 0.0001	43.8	89/90	
