# Supplementary figures and images for: Mutations in Nonessential eIF3k and eIF3l Genes Confer Lifespan Extension and Enhanced Resistance to ER Stress in Caenorhabditis elegans
Source: PLoS Genet. 2016 Sep 30;12(9):e1006326. doi: 10.1371/journal.pgen.1006326 (PMC5045169; doi:10.1371/journal.pgen.1006326)

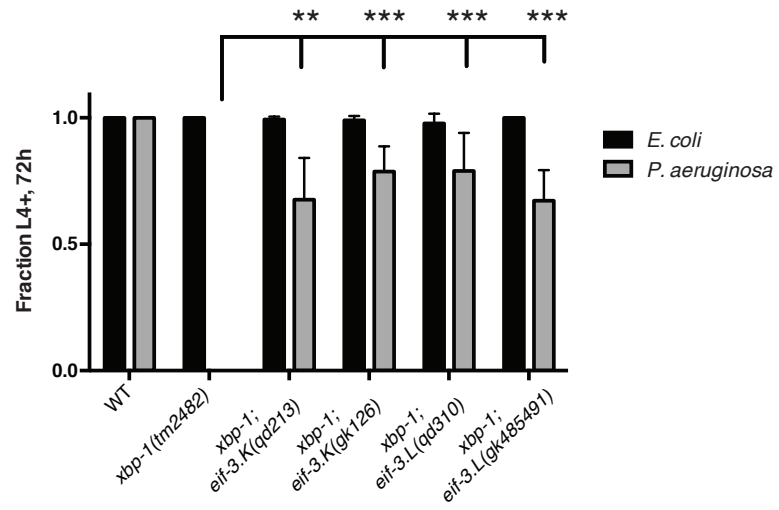

Supplement: S1 Fig — Development assay monitoring the growth and viability of the indicated genotypes on E. coli or P. aeruginosa at 25°C. 50–100 eggs were laid on each plate and following 72h the fraction reaching the L4 larval stage or older were counted. Error bars reflect the S.D. of 3 plates. A Student’s t-test was used to assess significance: **P<0.01, ***P < 0.001. (PDF) [file pgen.1006326.s003.pdf]

**A**

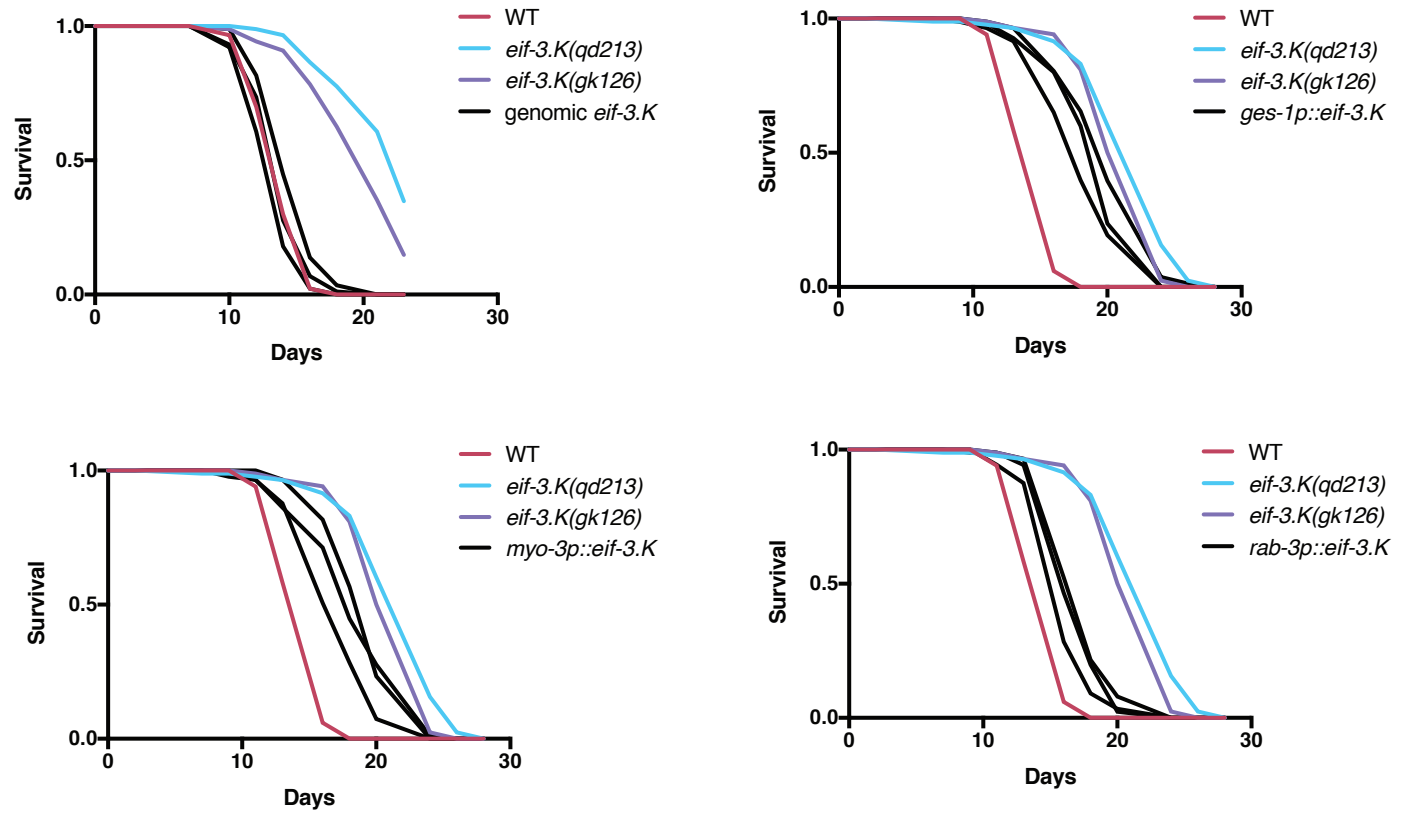

**B**

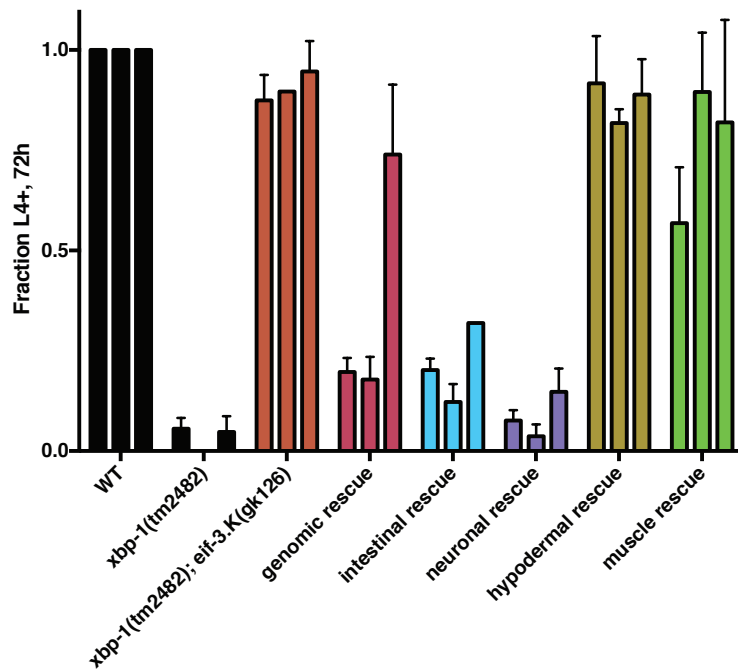

Supplement: S2 Fig — (A) Survival curves of the indicated genotypes at 25°C. Times indicated are days post-L4 stage. (B) Development assay monitoring the growth and viability of the indicated genotypes on P. aeruginosa at 25°C. 50–100 eggs were laid on each plate and following 72h the fraction reaching the L4 larval stage or older were counted. Error bars reflect the S.D. of 3 plates. (PDF) [file pgen.1006326.s004.pdf]
